# Metropolitan Association for Befriending Young Servants

**Published:** 1889-06-29

**Authors:** E. Henderson

**Affiliations:** 268, Liverpool-road, N.


### METROPOLITAN ASSOCIATION FOR BEFRIENDING YOUNG SERVANTS.

Sir,—Some time ago you were good enough to insert a letter in your valuable and always interesting paper which was the means of obtaining a home for a destitute girl. I feel therefore encouraged to seek your interest in the following case: A young woman, aged twenty-four, who, through an accident, has quite lost the use of her right hand and arm. Formerly she got her living as a servant; now that is quite out of the question, but she is quite able to take a situation as messenger or doorkeeper, or something of the kind, for small wages. It is a very sad case. She has not a relative in the world, and no home. She is very willing to work, and is a bright, quick, intelligent girl. I should be so grateful if you would kindly make this known.

268, Liverpool-road, N. E. Henderson.

